# Pharmacokinetics and Toxicity of Sodium Selenite in the Treatment of Patients with Carcinoma in a Phase I Clinical Trial: The SECAR Study

**DOI:** 10.3390/nu7064978

**Published:** 2015-06-19

**Authors:** Ola Brodin, Staffan Eksborg, Marita Wallenberg, Charlotte Asker-Hagelberg, Erik H. Larsen, Dag Mohlkert, Clara Lenneby-Helleday, Hans Jacobsson, Stig Linder, Sougat Misra, Mikael Björnstedt

**Affiliations:** 1 Department of Oncology, Karolinska Institutet, Karolinska University Hospital Södersjukhuset, SE-118 83 Stockholm, Sweden; E-Mail: clara.lenneby-helleday@karolinska.se; 2 Department of Women’s and Children’s Health, Karolinska Institutet, Karolinska University Hospital Solna, SE-171 76 Stockholm, Sweden; E-Mail: staffan.eksborg@karolinska.se; 3 Department of Laboratory Medicine, Division of Pathology F46, Karolinska Institutet, Karolinska University Hospital Huddinge, SE-141 86 Stockholm, Sweden; E-Mails: marita.wallenberg@ki.se (M.W.); sougat.misra@ki.se (S.M.); mikael.bjornstedt@ki.se (M.B.); 4 Medical Products Agency, P.O. Box 26, SE-751 03 Uppsala, Sweden; E-Mail: charlotte.asker-hagelberg@mpa.se; 5 Clinical Pharmacology Unit, Department of Medicine, Karolinska Institutet, Karolinska University Hospital, Stockholm, Solna, SE-171 76 Stockholm, Sweden; 6 National Food Institute, Technical University of Denmark, DK-2860 Søborg, Denmark; E-Mail: ehlar@food.dtu.dk; 7 Department of Radiology, Karolinska University Hospital Södersjukhuset, SE-171 76 Stockholm, Sweden; E-Mail: dag.mohlkert@sodersjukhuset.se; 8 Department of Radiology, Karolinska University Hospital, Solna, SE-171 76 Stockholm, Sweden; E-Mail: hans.jacobsson@karolinska.se; 9 Department of Oncology-Pathology, Karolinska Institutet, Stockholm SE-171 76, Sweden; E-Mail: Stig.Linder@ki.se; 10 Department of Medicine and Health, Linköping University, Linköping SE-581 83, Linköping, Sweden

**Keywords:** sodium selenite, carcinoma, pharmacokinetics, maximum tolerated dose

## Abstract

Background: Sodium selenite at high dose exerts antitumor effects and increases efficacy of cytostatic drugs in multiple preclinical malignancy models. We assessed the safety and efficacy of intravenous administered sodium selenite in cancer patients’ refractory to cytostatic drugs in a phase I trial. Patients received first line of chemotherapy following selenite treatment to investigate altered sensitivity to these drugs and preliminary assessment of any clinical benefits. Materials and Methods: Thirty-four patients with different therapy resistant tumors received iv sodium selenite daily for consecutive five days either for two weeks or four weeks. Each cohort consisted of at least three patients who received the same daily dose of selenite throughout the whole treatment. If 0/3 patients had dose-limiting toxicities (DLTs), the study proceeded to the next dose-level. If 2/3 had DLT, the dose was considered too high and if 1/3 had DLT, three more patients were included. Dose-escalation continued until the maximum tolerated dose (MTD) was reached. MTD was defined as the highest dose-level on which 0/3 or 1/6 patients experienced DLT. The primary endpoint was safety, dose-limiting toxic effects and the MTD of sodium selenite. The secondary endpoint was primary response evaluation. Results and Conclusion: MTD was defined as 10.2 mg/m^2^, with a calculated median plasma half-life of 18.25 h. The maximum plasma concentration of selenium from a single dose of selenite increased in a nonlinear pattern. The most common adverse events were fatigue, nausea, and cramps in fingers and legs. DLTs were acute, of short duration and reversible. Biomarkers for organ functions indicated no major systemic toxicity. In conclusion, sodium selenite is safe and tolerable when administered up to 10.2 mg/m^2^ under current protocol. Further development of the study is underway to determine if prolonged infusions might be a more effective treatment strategy.

## 1. Introduction

The micronutrient selenium is an essential trace element and confers its antioxidant properties upon incorporation into selenoproteins with oxidoreductase functionalities. However, redox active free selenium compounds at moderate to high doses are capable of perturbing cellular redox homeostasis upon generating ROS [1]. Increased ROS production exerts potent cytotoxic effects on proliferating cancer cells that exhibit lower threshold tolerance to ROS in comparison to normal cells [2,3]. Hence, ROS-producing agents, including redox active selenium compounds comprise a new class of cancer therapeutics targeting deregulated redox homeostasis in cancer cells. Specifically, high doses of sodium selenite exhibit promising antitumor activities as exemplified in many preclinical studies, both *in vitro* and *in vivo*. Pharmacodynamics data suggest that selenite targets several key cancer-associated signaling pathways and induces multimodal regulated cell death pathways [4,5,6,7]. Several studies have reported higher cytotoxicity of selenite to tumor cells compared to non-malignant cells at comparable dose [8,9,10], thereby presenting a plausible therapeutic window. We and others have previously shown that drug-resistant cancer cells are more sensitive to selenite than their drug-sensitive variants both *in vitro* [11,12,13] and in transplantable animal models [14]. In experimental carcinogenesis and transplantable tumor models, high dose of selenite prevents against the tumorigenesis and the progression of established carcinoma [15,16,17,18]. Together, the specificity of selenite to target cancer cells and especially those of drug-resistant variants highlights the key properties of selenite as a potent anticancer agent.

In spite of promising findings in a majority of these preclinical investigations, no systematic clinical studies have been carried out to evaluate the therapeutic potential of this compound in cancer patients. Two independent studies in the early twentieth century reported subjective improvement, prolonged life span and disease free survival in a fraction of patients with inoperable carcinoma following iv administration of colloidal selenium [19,20]. So far, selenite pharmacodynamics data are only available in sepsis patients with neither increased incidence of acute toxicity symptoms nor higher frequency of adverse events when administered with a bolus-loading dose of 2.0 mg Se followed by continuous infusion of 1.6 mg Se per day for 10 days [21]. Another study has reported similar finding on the incidences of adverse events following iv administration of comparable dose of selenite in sepsis patients [22]. However, lack of pertinent pharmacokinetics and safety data in dose-escalation studies and presumed high toxicity of selenite to humans have limited its potential application as a cancer therapeutic.

In view of the much anticipated investigation aiming at evaluating the efficacy of selenite in the treatment of cancer, the present phase I study was designed to evaluate the safety, establish the MTD of iv administered selenite and preliminary efficacy evaluation following termination of selenite treatment and subsequent treatment with cancer therapeutics in cancer patients with advanced disease. Based on the previous studies, we postulated that selenite might work as a remedy against cancer in three ways: the antitumor effect by itself, by reversing chemoresistance and by ameliorating toxic effects from chemotherapy. In accordance, each patient was treated with identical chemotherapy as their first line of treatment following the assessment of the selenite effect. This made it possible to compare the toxicity and primary anti-tumor effects before and after selenium administration.

## 2. Experimental Section

### 2.1. Patient Eligibility

Inclusion criteria comprised confirmed malignant disease with progressing tumor in spite of treatment with established drugs for respective diagnosis, capacity to cooperate, a good or fairly good performance status (WHO performance status 0–2), and age above 18 years. The exclusion criteria consisted of any other complicating disease interfering with the possibility to carry through the study and any other cancer treatment 3 weeks prior the treatment commencement. The study was approved by the Ethical Committee of Stockholm (2006/429-31/3), the Swedish Medical Products Agency and registered in EU Clinical Trial Register (Eudra CT Number: 2006-004076-13). All patients provided informed consent prior to receiving the treatments.

### 2.2. Study Design

**Figure 1:**
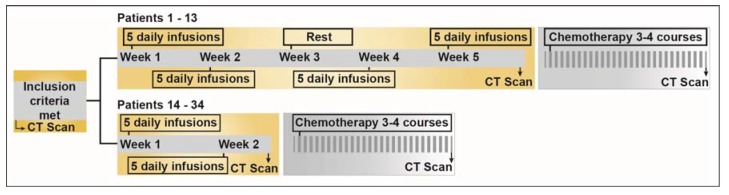
Schematic diagram depicting the study design. The first 13 patients were treated as per the initial study protocol that was amended and Patients 14–34 were subjected to new treatment schedule. For detailed information on the specific chemotherapeutic interventions in each patient, please refer to Supplementary Table S1. 160 × 41 mm (300 × 300 DPI).

This phase I trial was an open-label dose-escalation study of iv administered sodium selenite as a single agent. Detailed study design is presented in Figure 1 and dose escalation schedule is described in Table 1.

**Table 1:** Dose Exposure/Escalation Design.

Cohort No.	No. of Patients	Daily Dose (mg/m^2^)	Treatment Schedule	Total Median dose (mg); (range)
1	3	0.5	OPD; 5 d/week; 4 weeks	18.0 (17.5–18.0)
2	3	1.0	OPD; 5 d/week; 4 weeks	36.8 (34.0–42.0)
3	3	1.5	OPD; 5 d/week; 4 weeks	60.0 (50.0–62.0)
4	4	2.0	OPD; 5 d/week; 4 weeks	72.5 (66.5–92.0)
Protocol amendment; 50% dose escalation in subsequent cohorts				
5	4	3.0	OPD; 5 d/week; 2 weeks	49.0 (28.8–57.5)
6	3	4.5	OPD; 5 d/week; 2 weeks	74.0 (62.0–98.0)
7	3	6.8	OPD; 5 d/week; 2 weeks	117.0 (115.0–131.0)
8	3	10.2	OPD; 5 d/week; 2 weeks	214.0 (176.0–225.0)
9	6	15.3	OPD; 5 d/week; 2 weeks	77.7 (58.0–326.0)
10	2	12.8	OPD; 5 d/week; 2 weeks	159.8 (107.6–212.0)

Table 1: Dose is expressed as “total” selenium equivalent, not as selenite. One patient in cohort 3 received 19/20 treatments and another patient in cohort 4 received 5/10 treatments. At 15.3 mg/m^2^ dose, only one patient received all the planned treatment. Rest of the patients in this cohort received a total of 2–5 treatments (median value 3) following which treatment was stopped either due to onsets of grade 3–4 SAE or at patients’ own discretion. Note that cohort 10 received a dose of 12.8 mg/m^2^ to define whether MTD lies within an intermediate dose. An example: For a patient with surface area 1.72 m^2^ receiving 10.2 mg/m^2^ dose, the daily dose is equal to 17.544 mg (1.72 m^2^ × 10.2 mg/m^2^). Surface area calculation: √(height × weight/3600); where height is in cm and weight is in kg. [23]. Abbreviation: OPD—once per day.

Each cohort consisted of at least 3 patients who were treated at escalating dose levels according to a predefined schedule. The patients received the same daily dose of selenite throughout the whole treatment. If 0/3 patients had DLT, the study proceeded to the next dose-level. If 2/3 had DLT, the dose was considered too high and if 1/3 had DLT, 3 more patients were included. Dose-escalation continued until the MTD was reached. MTD was defined as the highest dose-level on which 0/3 or 1/6 patients experienced DLT. To further titrate the MTD, an intermediate dose was chosen in between the dose resulted in high DLT and the nearest lower dose with acceptable toxicity.

### 2.3. Drug, Dose Escalation Design and Infusion Schedule

GMP grade sodium selenite (Intro-Selen i.v., Pharma Nord ApS, Denmark) was used for the study. The first cohort was treated daily with 0.5 mg/m^2^ selenium, equivalent to 1.1 mg/m^2^ selenite, thus approximately a total dose of 0.9 mg selenium per day for a medium sized Swedish patient. The first 13 patients (first 4 cohorts) received 10 treatments during 2 weeks (no treatment during weekends). Following a week of rest, all the patients received the same treatment for another 2 weeks. The objectives of the prolonged treatment were to achieve a gradual increase of plasma selenium concentration and to detect any serious toxicity at the earliest. Initially, the dose escalation for a successive new cohort was 0.5 mg/m^2^. However, after initial plasma selenium levels measurement in the study cohorts receiving up to 2.0 mg/m^2^ dose, we found no or very limited increase in plasma selenium level in the 3rd and 4th week. Therefore, an amended protocol was adopted in which the treatment schedule was changed to 10 treatments during 2 weeks with a faster dose-escalation (50% dose escalation for each new cohort). Selenite was administered during 20 min in the first 21 patients. Since each vial comprised only 0.4 mg selenium in 10 mL, Patient 22 to Patient 29 received the treatment for 40 minutes to avoid too high infusion volumes at short duration. From patient 30 and later, the infusion was given during 4 h. Pretreatment with betametason and omeprazol were routinely given to patients at dose levels of 10.2 mg/m^2^ and above.

### 2.4. Toxicity Assessment

With a few exceptions, all patients visited OB before and after inclusion, at least once a week during the treatment, before each chemotherapy treatment and at the end-of-treatment. At these occasions, medical history was recorded and physical examination was performed. Patients underwent CT examination one week before the start, 3 days after the completion of selenite treatment and after the final chemotherapy treatment. From Patient 27, the first and second CT examinations were substituted with PET-CT examination. Routine blood parameters were analyzed before, during and after the treatment. ECG was examined before the start and after both the selenite and chemotherapy treatments.

### 2.5. Chemotherapy

All patients had earlier received 1 up to 4 lines of chemotherapy pertinent to the respective diagnosis. Detailed description of chemotherapy regimen for each patient is presented in supplementary Table S1. Progression in spite of any treatment was an inclusion criterion. We thought that it was of potential clinical interest to assess chemotherapy-induced toxicity after selenite treatment knowing that most of the patients experienced depleted bone marrow reserves after receiving 2–4 lines of chemotherapy. Consequently, being in significantly worse general condition compared with what was the case when receiving their first line of chemotherapy also rendered the patients more vulnerable to toxicity. Such assessment was performed by administering the same chemotherapy treatment following selenite treatment in each patient. This gave an opportunity to compare both the toxicity and effect of primary treatment from patient records and the subsequent chemotherapy regimen. It is important to note that the idea of implementing chemotherapy after the selenite treatment may not be comprehended as combined treatment strategy. Rather, this approach opened up the possibility to evaluate the modulatory effects of selenite treatment on toxicity arising out of subsequent chemotherapy and also to study the possible reversal of chemoresistance following selenite treatment.

### 2.6. Pharmacokinetic Evaluation

#### 2.6.1. Blood Sampling and Selenium Analysis

Blood samples were collected about 5 min prior and post infusion at all treatments. The plasma fractions were obtained by centrifugation and stored at –70 °C until analyses. Selenium (as ^82^Se) in the plasma samples was quantified by ICP-MS, as described previously [24] (for details, see data supplement).

#### 2.6.2. Pharmacokinetic Analyses

Pharmacokinetic analyses were performed by using the Win-Nonlin program Standard Edition version 1.5 (Pharsight Corporation, Mountain View, CA, USA). The one compartment infusion model was used for the evaluation of the pharmacokinetics. The reciprocal concentrations were used as weights for the iterative procedure. The output from the curve fitting program gives the area under the plasma selenium concentration–time curve (AUC) for a single dose, calculated from the fitted zero-time intercepts and exponential rate constants of a one exponential function giving AUC values from time zero to infinity. AUC values during the entire treatment period (*i.e.*, from day 0 to day 35) were evaluated by applying the trapezoidal rule to estimated time–concentration curves.

#### 2.6.3. Statistical Analyses

We used descriptive statistics (SigmaPlot, Version 11) to summarize the data for safety, toxicity, pharmacokinetics and clinical activity.

## 3. Results

### 3.1. Patients

A detailed description of patient characteristics is presented in Table 2. All the patients received at least two doses of selenite and were evaluable for toxicity. Four patients stopped treatment because of DLT and two patients chose to discontinue in spite of no DLT. For one patient with progressing liver metastases, treatment was stopped due to quickly rising bilirubin.

### 3.2. Dose Escalation, Toxicity and MTD

Of the first 13 patients included, 12 received all planned selenite treatments. One of the patients died from acute pneumonia with heart complications after 19 treatments. Of the following 21 patients, 13 received 10/10 planned selenite infusions. In a group of patients with advanced, progressing carcinoma and expected short survival, it was sometimes difficult to distinguish between the symptoms from the selenite treatment and symptoms from the progressing carcinoma, posing certain limitations on the side effects evaluations in the present study. There were hardly any symptoms of toxicity below 3.0 mg/m^2^ dose. The most common toxicities were nausea, vomiting and fatigue, observed at dose levels 4.5 mg/m^2^ and higher (Table 3). No DLT was observed at doses ≤10.2 mg/m^2^. At 15.3 mg/m^2^ dose-level, DLTs were observed in 2/6 patients and the dose was reduced to 12.8 mg/m^2^ in the next study cohort, according to the dose-escalation rules of the study. At this dose, both the first and second patient had dose limiting toxicity. Thus, the MTD was considered to be 10.2 mg/m^2^. The highest cumulative dose given without any serious adverse events was 326 mg selenium during 12 days with no evidence of DLT in a 66 years old patient, at dose level 15.3 mg/m^2^. One patient with a throat obstacle from a hypo-pharyngeal carcinoma experienced vomiting after three selenite treatments, causing aspiration pneumonia, leading to death within a few days (Table 3).

**Table 2:** Demographics and clinical characteristics of patients enrolled in SECAR trial.

Characteristic	Patients (*N* = 34)	
	No.	%
**Age, Years**		
Mean	59.7	
SD	11	
Median	61.5	
Range	37–79	
**Sex**		
Male	19	56
Female	15	44
**WHO performance status**		
0	9	26.5
1	19	55.9
2	6	17.6
**Primary tumor types**		
Non-small cell lung carcinoma	21	61.8
Small-cell lung carcinoma	3	8.8
Colon cancer	4	11.8
Rectal cancer	2	5.9
Malignant mesothelioma	1	2.9
Ethmoidal cancer	1	2.9
Tongue base cancer	1	2.9
Testicular teratoma	1	2.9
**Stages**		
Stage I	0	0
Stage II	0	0
Stage III	7	20.6
Stage IV	27	79.4
Refractory to first line therapy	34	100

Abbreviations: WHO—World Health Organization; SD—Standard Deviation.

**Table 3:** Treatment-Emergent Clinically Significant Adverse Events (AEs) by Preferred Terms.

Preferred Terms	Grades 1–2		Grades 3–4		Any Grade	
	No.	%	No.	%	No.	%
Alopecia	2	6	0	0	2	6
Anorexia	3	9	0	0	3	9
Anxiousness	0	0	1	3	1	3
Arthralgia	2	6	0	0	2	6
Chest pain	1	3	0	0	1	3
Confusion	0	0	1	3	1	3
Cramps in legs and/or finger	6	18	0	0	6	18
Dyspnea	2	6	1	3	3	9
ECG change	0	0	1	3	1	3
Fatigue	16	47	2	6	18	53
Garlic smell of breath	5	15	0	0	5	15
Gastritis	1	3	0	0	1	3
Hallucination	0	0	1	3	1	3
Hot Flush	1	3	0	0	1	3
Nausea	12	35	1	3	13	38
Pain at infusion site	2	6	0	0	2	6
Peripheral sensory and motor neuropathy	1	3	0	0	1	3
Pressure on thorax	0	0	1	3	1	3
Pruritus legs	1	3	0	0	1	3
Stomach pain	1	3	0	0	1	3
Stomach swelling	1	3	0	0	1	3
Thrombopenia	1	3	0	0	1	3
Unconsciousness	0	0	1	3	1	3
Vertigo	1	3	0	0	1	3
Vomiting	7	21	0	0	7	21

Note: Adverse events of grade ≥ 3 were only observed at dose ≥ 12.8 mg/m^2^.

**Figure 2:**
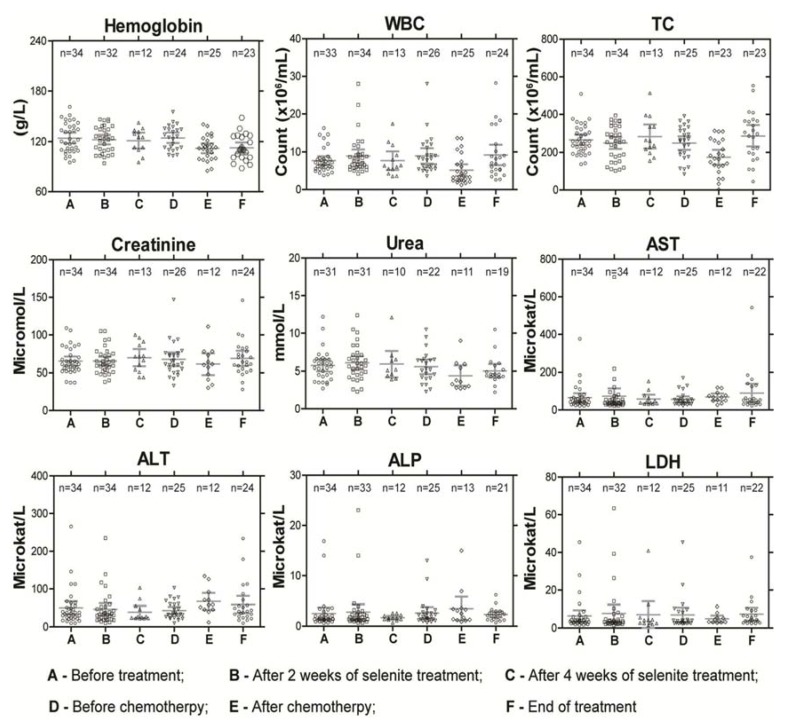
Routine blood parameters in patients during the course of the treatment as a measure of systemic toxicity. Abbreviations: WBC—white blood cell count; TC—thrombocytes count; AST—aspartate amino transferase; ALT—alanine amino transferase; ALP—alkaline phosphatase; and LDH—lactate dehydrogenase. 155 × 131mm (300 × 300 DPI).

The reasons to stop treatment in the four patients with selenite-induced DLT was confusion and hallucinations after four treatments in one patient at dose-level 15.3 mg/m^2^ due to possible interaction with a SSRI antidepressant drug (Mirtazapin); excessive fatigue and emesis after two treatments in another at the same dose-level; chest pain, anxiousness with signs of heart hypoxia on ECG and marginally increased Troponin T (but no myocardial infarction and normalized ECG within four days) in one patient; and unconsciousness, dyspnea and pronounced hypoxia with need of assisted breathing in the other, both at dose level 12.8 mg/m^2^ (Table 3). At higher doses, cramps of short duration in fingers and calves (similar to night cramps of the legs) and pain in the tumor were common. Garlic smell of breath was found in 15% of patients treated at dose levels of 3 mg/m^2^ and above. All toxicities except fatigue, a very subjective symptom, were reversible within one or two days. We did not observe any sequelae. There was no or very limited effect on routine blood tests (Figure 2). In a few patients above dose-level 6.8 mg/m^2^, a mild thrombocytopenia (TC count not below 80,000/mL) was found. Clinically most of the patients were euthyroid during the whole follow up period except in few patients with aberrant triiodthyronin and thyroxin values.

**Figure 3:**
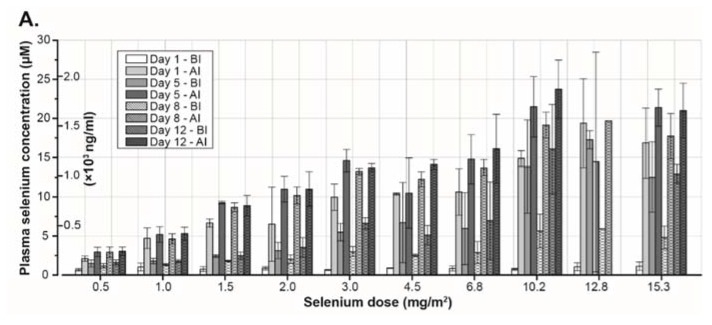

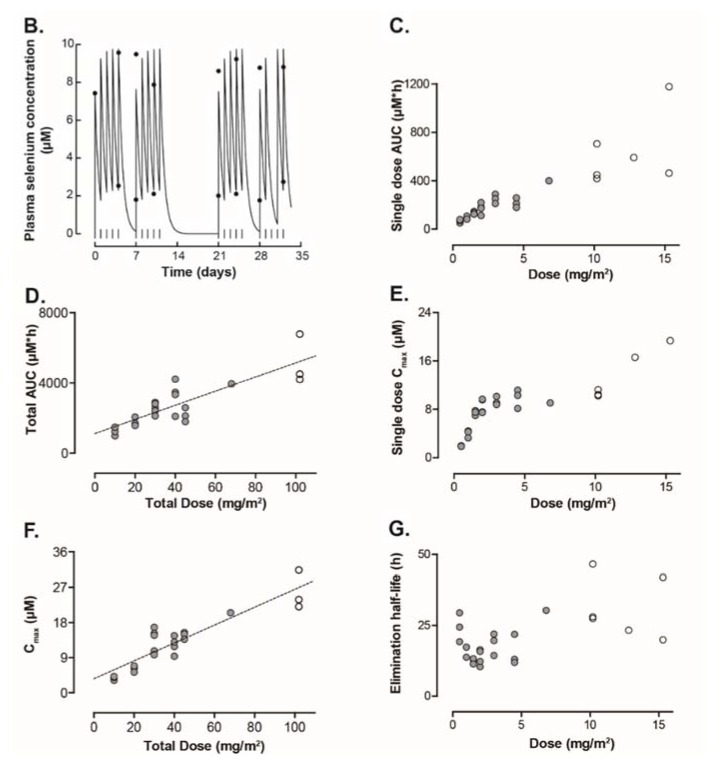
(**A**) Plasma selenium concentration in different cohorts receiving increasing concentration of sodium selenite during the first two-weeks of treatment. (**B**) Plasma selenium concentration–time curve in Patient 9. The daily dose (1.5 mg/m^2^) was given as a 20 min constant rate infusion. Start of infusions is indicated by the vertical lines. Measured serum concentrations are given by the filled circles. (**C**,**D**) Systemic selenium exposure is expressed as area under the serum concentration–time curve (µM*h). Figure 3C shows data from a single dose of selenite and data from the entire treatment period are presented in Figure 3D. (**E**,**F**) Figure 3E represent maximum concentration of selenium after a single dose of selenite. Maximum concentration of selenium during the entire treatment period is presented in Figure 3F. (**G**) Elimination half-life of selenium at different doses. In all the figures, filled circles indicate data from 20 min infusions and open circles data from 40 min infusions. Abbreviations: BI—before injection; AI—after injection; AUC—area under curve; and C_max_—maximum concentration 162 × 253mm (300 × 300 DPI).

### 3.3. Pharmacokinetics

The pharmacokinetic modeling was successful for data from all patients receiving all or most of the treatment. In eight patients who stopped early and in one patient who tested positive for HIV, there were no or not enough data to calculate any pharmacokinetic values. There was a close correlation between observed and predicted selenium concentration (correlation coefficient, *r* = 0.94 ± 0.05; mean ± standard deviation). The result from the pharmacokinetic curve fitting procedure using data from one patient is given in Figure 3B. There was a linear increase of AUC with the dose both for data estimated for a single dose of selenium (given by the output from the used pharmacokinetic curve fitting program) and for repeated administration (Figure 3C,D). The maximum plasma concentration (C_max_) from a single dose of selenium increased with the dose in a non-linear pattern (Figure 3E). In contrast, there was a linear increase of observed C_max_ of selenium during the entire study period with the total dose (Figure 3F). The median elimination half-life was 18.25 h (range 10.4–46.6 h) (Figure 3G).

### 3.4. Clinical Assessment

**Figure 4:**
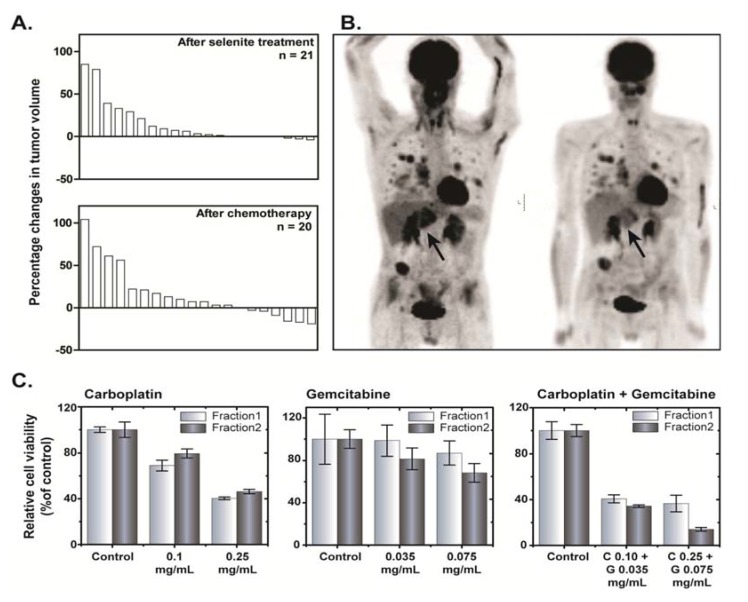
(**A**) Waterfall plot showing tumor response following selenite treatment (top panel) and subsequent administration of chemotherapeutic drugs (bottom panel). In the waterfall plot, a positive value indicates increase in tumor volume and *vice versa*. (**B**) Anterior-posterior Maximum Intensity Projection (MIP) PET images of a 48 year old man with multiple metastases from a carcinoma of the left sinus ethmoidalis, acquired 60 min after iv administration of [^18^F]-2-fluoro-2-deoxy-D-glucose (FDG) (4 MBq/kg body weight); left panel, baseline examination; and right panel, examination after 17 days following stopping his selenite treatment (three treatments with 28.2 mg selenite a day). A few hours after the first treatment, his primary tumor, localized behind his left eye, started to swell, producing a massive increase of his earlier modest exophthalmos. However, it returned to the earlier status after 1–2 days. This examination shows a general decrease of the FDG-uptake of the metastases, most evident in a large metastasis of the left liver lobe (arrow). (**C**) Toxicity of single or combined exposure of carboplatin and gemcitabine for 48 h to cells collected from pleural exudates from a patient. Fraction 1 indicates before selenite treatment and Fraction 2 indicates after selenite treatment. Data are presented as mean ± S.D. 160 × 143 mm (300 × 300 DPI).

The antitumor effect was a secondary endpoint. Any antitumor effect described herein should be interpreted with precaution due inherent uncertainty associated with the limited number of patients on each dose level. Tumor size (RECIST) was evaluated before and after the selenite treatment and following subsequent chemotherapy treatment. The results from these measurements are presented in the waterfall plot (Figure 4A), suggesting no consistent effect of selenite treatment on the tumor volume. None of the patients had a complete or partial response immediately after respective treatments but 13 patients had a stable disease after selenite treatment and 16 patients after subsequent chemotherapy. Patient 4, who received a stent in the trachea and main bronchus to get relieved from the risk of compression-associated breathing difficulties a week before starting selenite treatment, had a stable disease with gradually improved response during the following six months. The tumor was undetectable half a year later as revealed by CT and a PET-CT examination. She is still alive without any recurrent tumor for more than six years.

In six of the last patients included, a PET-CT examination with [^18^F]-2-fluoro-2-deoxy-d-glucose (FDG) was performed before and at the end of the selenite treatment. In one of these patients with testicular teratoma, SUV_max_ (Standardized Uptake Value for deoxyglucose in the tumor) increased after selenite treatment, indicating tumor-progression. In two patients (one with colon cancer and another with malignant mesothelioma) there were no changes. In three patients receiving 15.3 mg/m^2^, there were indications of a decreased uptake, albeit with some decreased uptake in the normal tissues in two of them. One patient with ethmoidal cancer (receiving 15.3 mg/m^2^), there was a clear decrease in SUV_max_, indicating a prominent decrease in liver metastasis burden (Figure 4B). In general, we also found out that change in plasma M65 (a measure of intact and caspase-cleaved cytokeratin 18) values from baseline was significantly lower in patients with stable disease following selenite treatment (Supplementary Figure S2).

In one patient with pleuritis, it was possible to address the modulatory effects of selenite treatment on the response of subsequent chemotherapy *ex vivo* following isolation of primary tumor cells from the pleura exudate. This was done before and after the selenite treatment. The cells were treated with carboplatin or gemcitabine and their combination *ex vivo.* The tumor cells were more sensitive to the combined carboplatin and gemcitabine treatment after the selenite treatment (Figure 4C). This patient had clinically and radiological determined tumor progression during the selenite treatment but regression when he was later treated with carboplatin and gemcitabine in combination.

Incidences of chemotherapy-induced toxicity were higher in 100% patients given less than 3 mg/m^2^ selenite in comparison to first line of treatment with the same drug(s) (supplementary Table S1). Therefore, it was registered as a need to decrease doses, prolong intervals between courses or to stop chemotherapy altogether. However, chemotherapy-induced toxicity was higher in only 46% of the patients when given above 3 mg/m^2^ doses of selenite.

## 4. Discussion

Selenium is known to be an essential micronutrient that can also elicit toxic effects in high concentrations. In this study, we report the first-in-human phase I trial to determine the MTD of iv administered sodium selenite in terminal cancer patients. One of the major aims of this study was to establish the safe dose level of selenite treatment. Sodium selenite was well tolerated in these patients at doses up to 10.2 mg/m^2^, therefore defined as MTD. At this dose and below, there were limited incidences of adverse events. Our observation was similar to what has been described earlier for selenite toxicity, mainly nausea, vomiting and fatigue—side effects that could be dose-limiting in spite of antiemetic treatment. The most significant adverse effects were heart toxicity with signs of ischemia, confusion, unconsciousness and impoverished breathing at dose level beyond MTD. Heart toxicity and breathing problems may be associated with negative impact of high dose of selenite on muscle contractility as had been earlier reported in perfused *ex vivo* heart and smooth muscles of rat and rabbit [25]. The dose limiting toxicity appeared some hours after selenium infusion, of short duration, not more than up to 6–8 h without any persisting toxicity or sequelae. Routine blood sample analyses indicated limited side-effects, transient in nature, if observed any. Notably, no bone marrow toxicity was evident.

The pharmacokinetic data suggested linear increase in plasma selenium concentration with respect to total dose. However, the highest peak concentration reached a plateau at dose level ≥10.2 mg/m^2^. Notably, variable infusion time at these dose levels provided a window for plasma clearance and thereby precluded any direct comparison. In one patient (dose level 12.3 mg/m^2^), the renal clearance was 46% of the total selenium dose administered, suggesting important role of kidney in excretion of selenium from selenite. At the end of study termination, the plasma selenium concentration returned to the baseline levels, indicating no apparent adverse effects of high dose of repeated selenite administration on physiological selenium homeostasis.

Selenite by itself was efficient in targeting certain tumors. However, a more pronounced effect was seen following subsequent chemotherapy in a subset of patients. From a clinical point of view, it is also known that single or combined use of cytostatic drugs might invoke tumor responses in certain patients after a time laps, although such phenomena are not common enough to justify repeated treatment with first line of treatment. Interestingly, many of the patients in our study responded again to their first line of chemotherapy, following which 13 patients were considered to have symptomatic benefit of the treatment. These *in vivo* effects corroborated well with the findings of the *ex vivo* study which demonstrated increased sensitivity towards identical chemotherapeutic drugs to patient-derived primary tumor cells. A recent study reported that selenite pre-treatment prevented the induction of carboplatin resistance *in vivo* in a nude mice model [26]. These results were in support of our findings and indicated that even if selenite in itself might be clinically useful in a subset of cancer patients; its subsequent use with chemotherapy might be therapeutically valuable.

Efficacy results of selenite treatment should be interpreted with caution because of the small number of patients enrolled and overall survival data included the effect of subsequent chemotherapy. Evidence for clinical benefits was observed following selenite treatment as reflected by stable disease in 38% patients. This group of patients had relatively stable plasma level of M65, reflecting what had been found out earlier in testicular cancer following chemotherapy [27]. However, it is important to note that these observations do not demonstrate favorable effects of selenite on overall survival with a limited number of patients. Albeit, it indicates that treatment with selenite and subsequent chemotherapy did not have a negative impact on survival. Two patients are still alive after six (CR) and one years, respectively. Besides, the median survival after the selenite treatment was 6.5 months, which might be considered a fairly long time in this group of treatment resistant, advanced tumors. Considering the advanced disease status and acquired resistance to the conventional chemotherapeutic treatments prior study enrollment in all patients, objective responses and stable disease in many patients are quite encouraging.

In general, tumor cells harbor high basal level of reactive oxygen species (ROS), associated with adaptive response to increased metabolic need for uninterrupted growth [3]. Our hypothesis was to treat cancer patients using high dose of selenite implicated in ROS generation within the margin of appreciable therapeutic index. Our data showed that certain tumors were selenite-resistant. The differences in tumor response to selenite treatment perhaps attributed to inter-tumor heterogeneity and their microenvironment that regulate selenium uptake at the target tissue. Context wise, the roles of reducing extracellular redox milieu and expression of cystine-glutamate antiporter xCT [24] (facilitate selenium uptake from selenite) in tumor tissues warrant further investigation as major determinants of successful chemotherapeutic application of selenite. One of the major limitations of the present study was the absence of any quantitative measure of selenium loading into the tumor. A significant number of patients (70%) had lung carcinoma that precluded the possibility of obtaining tumor biopsies without significant risks. It is also unclear whether the duration of infusion was sufficient enough to obtain maximal antitumor effect, given that the plasma half-life of selenite was relatively short. A steady-state plasma concentration of sufficient duration might be necessary to achieve efficient chemotherapeutic effects. While addressing this, we found out that a human lung cancer cell line is highly sensitive to selenite when exposed to low concentration for longer period of time, with concomitant low selenium accumulation and *vice versa* (see supplementary data, Figure S1). Thus, we plan to proceed with another phase I study of prolonged selenite infusion to determine MTD and clinical effects to find an optimal treatment schedule.

## 5. Conclusions

In conclusion, selenite might represent an interesting cancer therapeutic for the treatment of some tumors and might also be an efficient complement for cytostatic regimes. Our results defining MTD, safety, pharmacokinetics and clinical presentation provide essential information for further effect evaluating studies of selenite in the treatment of cancer. The findings from the study also indicate that selenite might work in the three ways as mentioned earlier—by itself, in combination with chemotherapy and as a remedy against chemotherapy toxicity. However, further studies are needed to define if it might be of value in the clinic and also which individual therapeutic aspect might represents the best therapeutic approach. But first, further studies are needed to find the optimal treatment schedule.

## Supplementary Files

Supplementary File 1
