# Supplementary material for: Pharmacokinetics and Toxicity of Sodium Selenite in the Treatment of Patients with Carcinoma in a Phase I Clinical Trial: The SECAR Study
Source: Nutrients. 2015 Jun 19;7(6):4978–94. doi: 10.3390/nu7064978 (PMC4488827; doi:10.3390/nu7064978)
Supplement: Supplementary File 1 [file nutrients-07-04978-s001.docx]

Supplementary Information

1. Isolation of Tumor Cells and Chemotherapy Drug Treatment

Pleura fluid was collected from a patient with pleuritis before receiving the first selenium injections and after the last injection. The fluid was centrifuged at 1100 rpm for 5 min. The cell pellets were washed several times with PBS and transferred to culture medium (Iscove’s Modified Dulbecco’s Medium, Sigma, Sweden) containing 20% heat inactivated foetal calf serum (FBS), 5% PEST and gentamycin (20 µg/mL) and cultured at 37 °C in a humidified atmosphere with 5% CO_2_. Since lot of inflammatory cells were present, the cells were washed with PBS regularly until only adherent cells remained in
the culture. When the cells started to proliferate, the FBS concentration was reduced to 10%. For the experimental set up, cells were counted and seeded into a 96-well plate (Sarstedts, Sweden) at density of 7000 cells/well and incubated for 48 h. Cells were washed carefully with PBS and treated with either Carboplatin or Gemcitabine, or in combination for 48 h. The cell viability was evaluated by XTT (Roche, Germany). All the cell experiments were completed between passages 2–4.

2. Selenite Toxicity to A549 Cells

A549 cells (ATCC) were seeded at a density of 1 × 10^5^ cells/mL in T-75 flasks (Sarstedts, Sweden) and maintained for 24 h prior selenite exposure. Cells were washed once with PBS and exposed to sodium selenite spiked in the medium. Following exposure termination, cells were washed twice with excess of PBS and trypsinized. The detached cells were again with ice cold PBS for few times and divided into two aliquots for protein and selenium measurement. For toxicity experiment, these cells were cultured in 96-well plate at a density of 5 × 10^4^ cells in 100 µL volume. Prior selenite exposure, cells were washed once with PBS and exposed to different selenite-spiked media for 24 h. The cell viability was measured by WST-1 (Roche, Germany) kit.

3. Measurement of Cytokeratin 18

Caspase cleaved cytokeratin 18 (reflecting apoptosis, M30) and total cytokeratin 18 (cleaved and whole, the latter reflecting necrosis, M65) were measured in plasma by using a commercial sandwich quantitative enzyme-linked immunosorbent assay (ELISA); the Peviva M65^®^ ELISA (VLVBio AB, Sundbyberg, Sweden), according to protocol of the manufacturer. All experiments were performed blindly to clinical correlates. The protein levels of CK18 were expressed as U/L.

4. Quality Control for Selenium Measurements

The relative standard deviation was 3.8% for samples containing 50 ng·Se/mL or higher. The accuracy of the analyses was assured by analyzing the Se concentration in the certified reference material (CRM) BCR 637 Human Serum in parallel with the study samples. The values found for the CRM
(89 ± 1 ng·Se/mL) did not deviate significantly from the certified value (80 ± 7 ng·Se/mL).


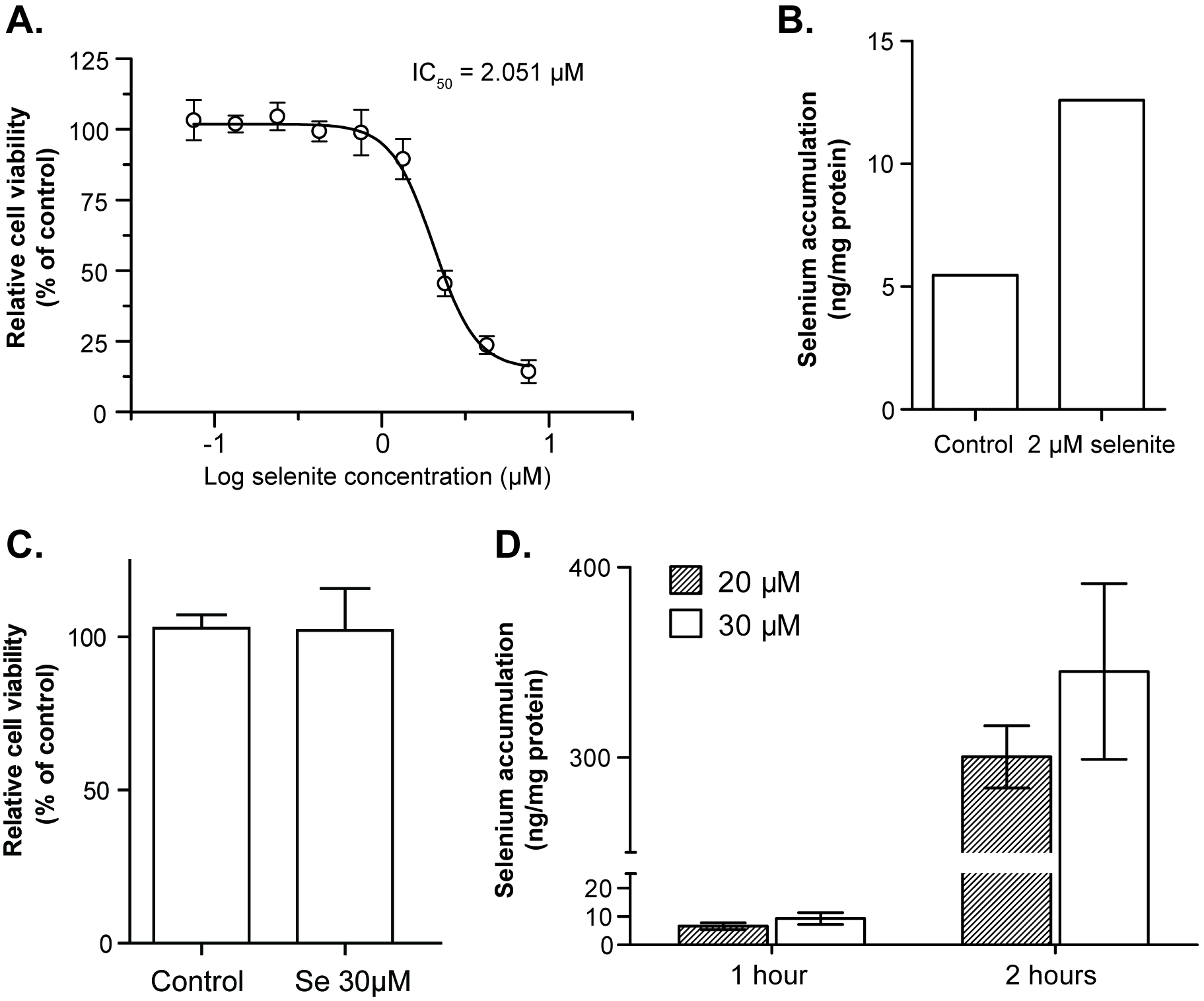


**Figure S1.** Cytotoxicity and uptake of selenite in A549 cells. (**A**) The calculated 51 h IC_50_ value for selenite was 2.051 µM (95% confidence interval 1.90–2.21 µM, *n* = 4). The data are presented as mean ± S.D. of “n” independent observations; (**B**) Selenium accumulation in these cells following 51 h exposure to 2.0 µM selenite; (**C**) A549 cells were exposed to 30 µM selenite for 2 h, washed with PBS and subsequently cultured for another 51 h in fresh medium. The data show relative cell viability (*n* = 3) after 51 h; (**D**) Selenium accumulation in A549 cells following exposure to 20 and 30 µM selenite for either 1 or 2 h (*n* = 3).


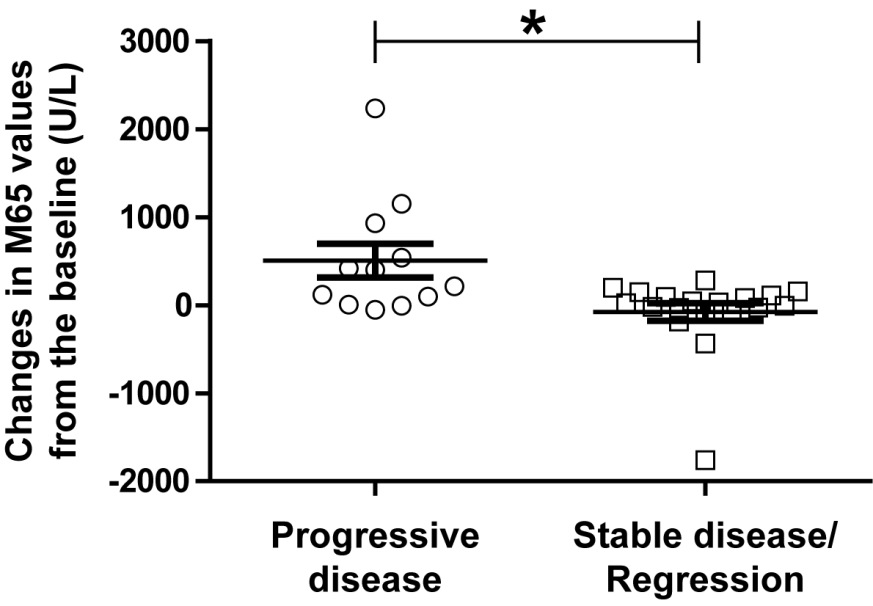


**Figure S2.** Comparison of changes in the plasma M65 fragments level following selenite treatment in patients. Data indicate changes of M65 values before and at the end of selenite treatment prior to receiving subsequent chemotherapy. There were significantly (*p* = 0.006, two-tailed unpaired *t*-test) lower M65 values in the responders when compared to patients with progressive disease. A cut off value of ± 2500 U/L was taken for the data analyses.

**Table S1.** A summary of chemotherapeutic interventions in the study group and the evaluation of toxicity symptoms arising out of chemotherapy before and after selenite treatment.

| **Patient No.** | **Cohort No.** | **Chemotherapy** | | **Toxicity** | |
| --- | --- | --- | --- | --- | --- |
|  |  | **First Line** | **After Selenite** | **First Line** | **After Selenite** |
| 1 | 1 | Gemcitabine + Carboplatin | Gemcitabine + Carboplatin | + | +++ |
| 2 |  | Etoposide + Carboplatin | Etoposide + Carboplatin | + | ++ |
| 3 |  | Gemcitabine + Carboplatin | Gemcitabine + Carboplatin | + | ++ |
| 4 | 2 | Gemcitabine + Carboplatin | Gemcitabine + Carboplatin | + | ++ |
| 5 |  | Gemcitabine + Carboplatin | Gemcitabine + Carboplatin | + | ++ |
| 6 |  | Gemcitabine + Carboplatin | Gemcitabine + Carboplatin | + | +++ |
| 7 | 3 | Gemcitabine + Carboplatin | Gemcitabine | ++ | +++ |
| 8 |  | Gemcitabine + Carboplatin | NC | NE | |
| 9 |  | Gemcitabine + Carboplatin | Gemcitabine + Carboplatin | + | ++ |
| 10 | 4 | Etoposide + Carboplatin | NC | NE | |
| 11 |  | Etoposide + Carboplatin | Etoposide + Carboplatin | + | +++ |
| 12 |  | Gemcitabine + Carboplatin | NC | NE | |
| 13 |  | Gemcitabine + Carboplatin | Gemcitabine + Carboplatin | + | ++ |
| 14 | 5 | Docetaxel + Cisplatin * | Docetaxel + Cisplatin | ++ | ++ |
| 15 |  | Gemcitabine + Carboplatin | Gemcitabine + Carboplatin | ++ | + |
| 16 |  | Gemcitabine + Carboplatin | NC | NE |  |
| 17 |  | Gemcitabine + Carboplatin | Gemcitabine + Carboplatin | + | + |
| 18 | 6 | Docetaxel + Cisplatin | Docetaxel + Cisplatin | ++ | ++ |
| 19 |  | Gemcitabine + Carboplatin | Gemcitabine + Carboplatin | + | + |
| 20 |  | Gemcitabine + Carboplatin | Gemcitabine + Carboplatin | + | ++ |
| 21 | 7 | 5FU | 5FU | + | ++ |
| 22 |  | Gemcitabine + Carboplatin | Gemcitabine + Carboplatin | + | +++ |
| 23 |  | Gemcitabine + Carboplatin | Gemcitabine + Carboplatin | + | ++ |
| 24 | 8 | 5FU + Oxaliplatin | 5FU + Oxaliplatin | + | + |
| 25 |  | 5FU + Oxaliplatin | 5FU | ++ | +++ |
| 26 |  | Pemetrexed + Carboplatin | Pemetrexed + Carboplatin | + | + |
| 27 | 9 | 5FU + Oxaliplatin | 5FU | + | +++ |
| 28 |  | 5FU + Oxaliplatin + Cetuximab | NC | NE | |
| 29 |  | 5FU + Irinotecan | 5FU + Irinotecan | ++ | + |
| 30 |  | Pemetrexed + Carboplatin | Pemetrexed + Carboplatin | + | + |
| 31 |  | Ifosfamide + Paclitaxel+ Cisplatin | NC | NE | |
| 32 |  | 5FU + Docetaxel + Cisplatin | NC | NE | |
| 33 | 10 | 5FU + Irinotecan | 5FU + Irinotecan | + | ++ |
| 34 |  | Docetaxel + Erlotinib | Docetaxel + Erlotinib | ++ | ++ |

Note: * Patient 14 received carboplatin twice as first line therapy. Abbreviations: NC—No chemotherapy,
NE—Not evaluable.

© 2015 by the authors; licensee MDPI, Basel, Switzerland. This article is an open access article distributed under the terms and conditions of the Creative Commons Attribution license (http://creativecommons.org/licenses/by/4.0/).
